# Ferulic acid alleviates retinal neovascularization by modulating microglia/macrophage polarization through the ROS/NF-κB axis

**DOI:** 10.3389/fimmu.2022.976729

**Published:** 2022-09-02

**Authors:** Xiaowei Sun, Lusheng Ma, Xiao Li, Jiao Wang, Yuanbin Li, Zijing Huang

**Affiliations:** ^1^ Department of Ophthalmology, The Affiliated Yantai Yuhuangding Hospital of Qingdao University, Yantai, China; ^2^ Department of Ophthalmology, The First Affiliated Hospital of Zhengzhou University, Zhengzhou, China; ^3^ Joint Shantou International Eye Center of Shantou University and The Chinese University of Hong Kong, Shantou, China

**Keywords:** retinal neovascularization, Ferulic acid, microglia/macrophage polarization, inflammation, NF-κB

## Abstract

Inflammation plays a pivotal role in ischemia-induced retinal neovascularization. Targeting microglia/macrophage-based neuroinflammation presents a promising therapeutic strategy. Ferulic acid (FA), a natural and active ingredient in plants, exerts favorable anti-oxidative and anti-inflammatory activities. In this study, we investigated the inhibitory effect of FA against hypoxia-induced retinal angiogenesis using cultured retinal vascular endothelial cells and an oxygen-induced retinopathy mouse (OIR) model. The immunoregulatory effect of FA on microglia/macrophage polarization was evaluated by detecting the expression of specific markers for both pro-inflammatory “M1” and anti-inflammatory “M2” phenotypes using co-immunostaining and polymerase chain reaction assays. The underlying molecular mechanism upon FA treatment was also explored. The results showed that FA supplement markedly inhibited retinal pathological angiogenesis both *in vivo* and *in vitro*. In addition, FA switched microglia/macrophage polarization from “M1” towards “M2” phenotype and alleviated the inflammatory response. Mechanically, the anti-angiogenic and anti-inflammatory properties of FA were mainly due to blockade of the ROS/NF-κB pathway. Our data demonstrated an anti-angiogenic effect of FA through regulating M1-to-M2 microglia/macrophage polarization, suggesting a potential therapeutic strategy for retinal neovascular diseases.

## Introduction

Ischemia/hypoxia-induced pathological retinal angiogenesis, also called as neovascularization, is the hallmark of several vision-threatening diseases, including retinopathy of prematurity, diabetic retinopathy, and retinal vein occlusion (1). Currently, targeting retinal neovascularization and macular edema using intravitreal injection of anti-vascular endothelial growth factor (VEGF) agents has achieved favorable clinical outcomes (2). However, there is a significant proportion of patients being resistant or do not respond to anti-VEGF treatments (3). Development of VEGF-independent anti-angiogenic molecules is urgently desired.

Microglia, the long living resident immune cells in the retina, play an important role in organizing developmental blood vessel formation (4). Microglia are a component of the perivascular glia limitans that bi-directionally interact with retinal endothelial cells and facilitate their subtle modulation in sprouting, migration, and refinement of retinal vascular systems (5). On the other hand, microglia could be highly activated and migrate towards the blood vessels during pathological conditions and are responsible for re-shaping the vasculacture (6). Like macrophage, microglia are recognized as highly plastic cells that activated ones adopt different phenotypes depending on the micro-environmental signals. In the brain, microglia/macrophage engage different functional programs through polarization towards an “M1” or “M2” phenotype. The M1 is considered pro-inflammatory and detrimental, characterized by the production of pro-inflammatory mediators including interleukin (IL)-1β, IL-6, tumor necrosis factor (TNF)-α, and CC chemokine ligand (CCL)-2, as well as an overexpression of inducible nitric oxide synthase (iNOS). Alternatively, microglia/macrophage assume an M2 phenotype with anti-inflammatory, immunosuppressive, and reparative properties that release cytokines including IL-10, transforming growth factor (TGF)-β, and others (7–9). Increasing evidence has demonstrated the role of neuroinflammation as an important pathophysiological event in the development of pathological angiogenesis. Identification of microglia/macrophage polarization in retinal neovascularization, together with a better understanding of the molecular mechanisms and polarization-based therapies, are therefore warranted.

Ferulic acid (FA) is a phenolic compound widely present in the plant cell walls (10). In recent years, FA has received increasing attention in the field of pharmaceutical, food, and cosmetics industry. It has low toxicity and possesses multiple pharmacological properties, including anti-oxidation, anti-inflammation, anti-thrombosis, and anti-carcinogenesis (11). Sustained FA supplement has been showed to inhibit microglia-mediated neuroinflammation in animal models of Alzheimer’s and Parkinson’s diseases (12), presenting a great prospect for the treatment of neurodegenerative disorders. FA could also attenuate hereditary retinal degeneration in mice by suppressing microglia-mediated inflammation (13). The anti-angiogenic effect of FA in ischemic retinopathy *via* targeting microglia/macrophage activity is hypothesized.

In this study, we aim to clarify the involvement of microglia/macrophage polarization in retinal neovascularization, to investigate the inhibitory effect of FA against retinal neovascularization using *in vivo* and *in vitro* experiments, and to explore the underlying molecular mechanisms.

## Materials and methods

### Oxygen-induced retinopathy murine model and treatment

C57BL/6J mice were purchased from Vital River Laboratories and kept in a specific pathogen-free facility in the Animal Laboratories of Yantai Yuhuangding Hospital. The OIR model was established according to a previously described method (14). Briefly, mouse pups and their nursing mother were exposed to 75% oxygen from postnatal day 7 (P7) to P12 using an Oxy Cycler system (BioSpherix, Inc., NY, USA) and then returned to room air. For treatment, FA (50 mg/kg, Sigma, No.1270311) or vehicle (phosphate buffered saline, PBS) were intragastrically administered twice daily from postnatal (P)12 to P17. Before intragastric administration, the mice were anesthetized with isoflurane in an animal anesthesia ventilator system (Matrx, America) to ensure that the mice experienced no pain and awoke quickly. For mechanistic investigation, lipopolysaccharide (LPS, 1mg/kg) was injected intraperitoneally at P14 to induce NF-κB activation. Neovascularization and microglia/macrophage polarization were evaluated on P17 in the OIR.

### Cell culture and treatments

The BV2 murine microglial cell lines were purchased from Kunming Institute of Zoology, Chinese Academy of Sciences, China. The cells were seeded into a 24-well plate with 1×10^5^ cells/well, and culture medium was Dulbecco’s Modified Eagle’s Medium (DMEM) supplemented with 10% FBS, streptomycin (50 mg/ml), and penicillin (50 U/L). For hypoxic stimulus, BV2 cells were transferred to hypoxic environments (1.5% O_2_), which was produced in a Forma 3111 Series II Water Jacketed Incubator (Thermo Fisher Scientific; Waltham, MA, USA) perfused with continuous flow of a mixture of 95% N_2_/5% CO_2_ gas. For treatments, FA was added to the culture medium with a final concentration of 0.5mg/ml at 6 hours after anoxic stimuli and lasted for 18 hours before further analysis. Human retinal endothelial cells (HRECs) were purchased from Procell Life Science&Technology (Wuhan, China). HRECs were grown in poly-L-lysine-coated plate in DMEM supplemented with 5% fetal FBS, streptomycin (50 mg/ml), and penicillin (50 U/L), endothelial cell growth factor, heparin, and hydrocortisone in a 5% CO_2_-enriched atmosphere with constant humidity. HRECs were grown in the 50 mM glucose medium (Sigma-Aldrich) for 48 h. D-Mannitol was used as an osmotic control. All experiments were performed by using passage 5 HRECs.

### Reactive oxygen species measurements

BV2 cells were cultured under hypoxic condition (1.5% O_2_). ROS-sensitive probe 2’,7’-dichlorodi-hydrofluorescein diacetate (DCFH-DA, MedChemExpress) was used to detect ROS levels. DCFH-DA has no fluorescence and can freely cross the cell membrane. After entering the cell, it can be hydrolyzed by intracellular esterase to generate DCFH, which cannot penetrate the cell membrane, and can be oxidized by the intracellular ROS to generate fluorescent DCF. BV2 cells were incubated with 5 µM staining solution in PBS in the dark for 30 min at 37°C, then washed with PBS twice, and then harvested with 0.05% trypsin-EDTA solution, suspended in 100μl PBS, and immediately analyzed with fluorescence microplate instrument with excitation wavelength 488nm, emission wavelength 525nm (Varioskan LUX, Thermo Fisher Scientific). The results were expressed as percentage of change in fluorescence and the control group was taken as 100%. Each of the freshly excised retina from OIR and control mice was incubated at 37°C in vials with 5 µM DCFH-DA. After 30 min of incubations, the retinas were washed and then sonicated in 100 µL PBS. The retinal homogenate was centrifuged, and 100 µL supernatant immediately assayed fluorometrically at excitation and emission wavelengths of 488 and 525 nm, respectively. The level of ROS in the retina was presented as fluorescence units/mg protein.

### Wound healing assay

BV2 microglial cells were treated with FA (0.5mg/ml) under hypoxia condition (1.5% O_2_) for 1 day. Cell supernatants were harvested and used as culture medium for HRECs. HRECs cells were then seeded in a 6-well plate (1×10^5^/well). When the cells merged into a monolayer, a sterile 200 µl pipette tip was used to make a line-shaped scratch at the surface of the cell layer. The degree of scratch healing reflecting cell migration was observed and images were captured in each group at 0 and 16h using a Leica DM4000 automated upright microscope system. The wound closure rate was calculated as: (initial distance-16h scratch distance/initial distance) ×100%, using Image-Pro Plus 6.0 (Media Cybernetic, MD).

### Tube formation assay

HRECs were starved for 24 hours, harvested by trypsin detachment, seeded at a density of 5000 cells/well in 24-well plates precoated with Matrigel, and were treated with FA (0.5mg/ml) just before being incubated in medium supplemented with 10 ng/mL VEGF. The images were taken using an inverted light microscope. The extent of tube formation was quantified by counting the number of meshes using Image J software (NIH, Bethesda, MD).

### Quantification of neovascularization in flat-mounted retinas

Eyes immersed in 4% paraformaldehyde (PFA) fixative for 30 min. Retinas were removed carefully and incubated with Isolectin B4 (1:50, Invitrogen, Shanghai, Co. Ltd.) for 1 hour at room temperature. Retinas were washed, cut into four pieces, and flat-mounted on the slides. The images were obtained using a microscope (Leica DM4000, Germany). Areas of retinal neovascularization were analyzed using Adobe Photoshop 2015 CC software (Adobe Systems, San Jose, CA) as previously described (14).

### Hematoxylin-Eosin staining and assessment of neovascular cells

Eyes were embedded in paraffin and cut into 5μm vertical slices. Retinal sections were washed and stained with hematoxylin buffer for 10 min at room temperature. The sections were rinsed in deionized water and then dipped in 1% Eosin solution for 15 sec. After rehydrated in alcohol gradient, slices were washed again and mounted. Histological analyses of retinal tissues were observed under a microscope (Leica DM4000, Germany) for calculating neovascular nuclei beyond the internal limiting membrane. Six eyes from 6 mice in each group were analyzed, and the experiments were independently repeated 3 times. For each eye, three horizontal sections were randomly chosen and a mean number of neovascular nuclei per section was determined. Neovascular cell nuclei protruding into the vitreous from the internal limiting membrane on the entire section were counted using a masked protocol.

### Immunofluorescence assay

For retinal wholemounts, eyes were immersed in 4% paraformaldehyde for 30 min, and the retinal cups were separated carefully from the eyeballs. Both retinal wholemounts and cell slides were stained using primary and secondary antibodies, washed extensively and flat-mounted. To detect microglia polarization, the following primary antibodies were used: anti-iba1 (Wako Chemicals), anti-iNOS (Santa Cruz), and anti-Arg1 (Santa Cruz). Secondary antibodies include Alexa Fluor 488-conjugated donkey anti-rabbit IgG H&L (Abcam) and Alexa Fluor 555-conjugated donkey anti-goat IgG H&L (Abcam). Retinal vessels were stained using Isolectin-B4 (Invitrogen, Shanghai, Co. Ltd.). The retinal wholemounts and cell slides were visualized *via* confocal microscopy (Carl Zeiss LSM710, Germany) and Leica DM4000 automated upright microscope system. Neovascularization and avascular area were analyzed using Image-Pro Plus 6.0 (Media Cybernetic, MD).

### TUNEL assay

Eyes were fixed in 4% paraformaldehyde for 30 min before cryoprotected in Tissue-Tek (Sakura Finetek USA Inc.) at -20°C. Eight-micrometer retinal cryosections were prepared. TUNEL staining (*In Situ* Cell Death Detection Kit, Fluorescein; Roche, Indianapolis, IN) was performed according to the manufacturer’s instructions. Retinal cryosections were stained with DAPI for 5 min at room temperature and visualized using confocal microscopy (Carl Zeiss LSM710).

### Quantitative reverse transcription polymerase chain reaction

The mRNA levels of iNOS, Arg1, CD86, CD206, IL-10, IL-6 and TNF-α were detected by qRT-PCR. The total RNA of the BV2 cells and retinas were extracted with TRIzol (Invitrogen) and converted into first-strand cDNA using random hexamer primers and the Reverse Transcriptase Superscript II Kit (Invitrogen) according to the manufacturer’s instructions. qRT-PCR was performed in a total volume of 20μL containing 2μL of cDNA, 10μL of 2×SYBR Premix Ex Taq, and 10μmol/L of the primer pairs. The PCR amplification protocols consisted of 95°C for 30s and up to 40 cycles of 95°C for 5s and 60°C for 34s according to the manufacturer’s instructions. The primers were *inos* fwd 5′-TTCAGTATCACAACCTCAGCAAG-3′ and rev 5′- T GGACCTGCAAGTTAAAATCCC-3′, *arg1* fwd 5′-TGGACAGACTA GGAATTGGCA-3′ and rev 5′-CCAGTCCGTCAACATCAAAACT-3′, *tnf-α* fwd 5′-GAGGCCAA GCCCTGGTATG-3′ and rev 5′-CGGGCCGATTGATCTCAGC-3′, *il-6* fwd 5′-ACT CACCTCTTCAGAACGAATTG-3′ and rev 5′-CCATCTTTGGAAGG TTCAGGTT G -3′, *cd86* fwd 5′-TCAATGGGACTGCATATCTGCC-3′ and rev 5′-GCCAAAATACTACCAGCTCACT-3′, *cd206* fwd 5′-GGGTTGCTATCACTCTCT ATGC-3′ and rev 5′-TTTCTTGTCTGTTGCCGTAGTT-3′, and *il-10* fwd 5′-GCTCTTACTGACTGGCATGAG-3′ and rev 5′-CGCAGCTCTAGGAGCATGTG-3′. *Gapdh* was used as a reference gene.

### Western blotting

Retinal protein was harvested and homogenized in lysis buffer (RIPA, Biocolors, Shanghai, China) containing protease and phosphatase inhibitor mini tablets (Thermo Fisher Scientific). The protein concentration was determined with bicinchoninic acid protein assay. Equal amounts of protein were used and western blotting was performed. Primary antibodies included anti-NF-κB (p65) (Cell Signaling Technology, Beverly, MA), anti-IL-6 (Abcam, Cambridge, MA), anti-IL-10 (Abcam), anti-TNF-α (Abcam), and anti-β-actin antibodies (Abcam). The gray intensity of proteins was measured using Image-Pro Plus 6.0.

### Evaluation of systemic side effects of FA

To assess the systemic side effects of FA, the body weights of mice were measured daily from P12 to P17, and vital organs (liver, kidney, brain) were weighed after the mice were euthanized. For histopathologic analysis, vital organs (liver, kidney, brain) were dissected in each group and fixed with 4% paraformaldehyde. Tissue sections were cut into 5 mm thick and Hematoxylin-Eosin staining was performed to detect any morphological alteration after FA treatment.

### Statistical analysis

For section analysis, 6 retinas from 6 mice in each group were used. Three sections from each retina were randomly chosen and a mean value was determined. The entire section was used for analysis, including TUNEL positive cell counting and neovascular cell counting on HE staining. For flatmount analysis, 10 retinas from 10 mice in each group were used for quantification of neovascular tufts. For immunostaining assays, 3 images were obtained in different locations of the retina (central, mid-peripheral and peripheral areas) and 6 retinas in each group were used for analysis. All experiments including Isolectin-B4 staining, PCR, immunofluorescence staining, flow cytometry and western blotting were independently repeated at least 3 times. Data are presented as mean ± standard error of measurement (SEM). Data were analyzed statistically using one-way ANOVA or 2-tailed Student *t* test, and *P* values < 0.05 were considered statistically significant.

## Results

### Microglia/macrophage underwent M1 polarization during retinal neovascularization

**Figure 1:**
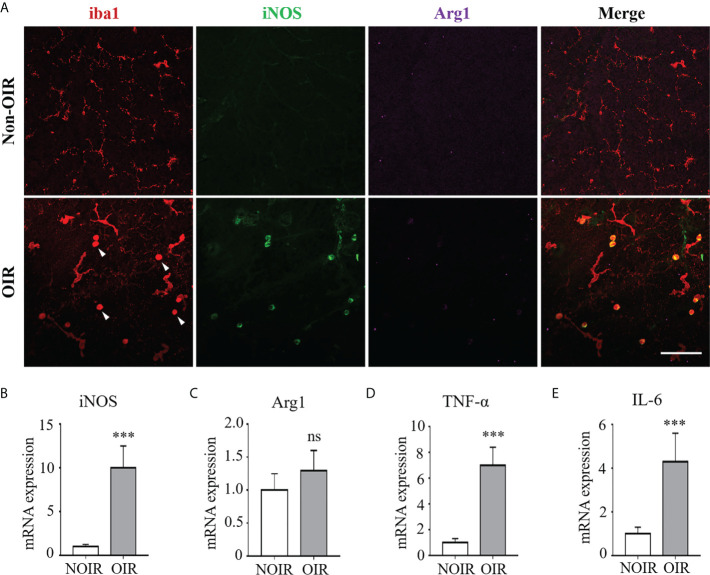
M1 microglia/macrophage polarization with increased inflammatory responses in retinal neovascularization. Microglia/macrophage polarization were evaluated on P17 in the OIR. **(A)** Representative images of immunofluorescence staining on retinal whole-mounts showed iba1^+^ microglia/macrophages were activated and featured as large cell body with few branches in the OIR retina (arrow heads). The activated microglia/macrophages presented distinct co-labeling of iNOS (M1 marker). Instead, there was few staining of Arg1 (M2 marker) in both OIR retina and non-OIR control. Scale bar, 100 μm. **(B, C)** Real-time PCR showed that mRNA level of iNOS **(B)** was increased while Arg1 **(C)** was insignificantly changed in the OIR compared with non-OIR control. **(D, E)** Hypoxia insults increased the mRNA levels of pro-inflammatory cytokines including TNF-α **(D)** and IL-6 **(E)**. Data are presented as mean ± SEM. n = 6 mice. ***P <0.001, ns, no significance.

Microglia/macrophage have been shown to possess different phenotypes under ischemic insults, of which a proinflammatory “M1” and an anti-inflammatory “M2” are the two classic polarization states (15). We evaluated microglia/macrophage polarization during experimental retinal neovascularization Microglia/macrophage polarization was evaluated on P17 of the OIR model in which neovascularization was maximal (**Supplementary Figure 1**). As shown in **Figure 1A**, activated microglia/macrophage exhibited an amoeboid-like morphology, presenting with swollen bodies and stubby branches, as compared with the resting ones in the non-OIR retinas resembling an octopus shape with small bodies and long branches. Of note, almost all activated microglia/macrophage in the OIR retinas were co-labeled with iNOS, a well-known M1 marker, whereas arginase1 (Arg1), a marker for M2, presented low immunoreactivity in both non-OIR and OIR retinas, indicating that activated microglia/macrophage mainly switched to M1 rather than M2 during retinal neovascularization (**Figure 1A**). We also investigated the RNA levels of iNOS and Arg1 in the OIR retinas. Consistently, the expression of iNOS was significantly upregulated (**Figure 1B**) while Arg1 had no significant change during the neovascularization phase as compared to non-OIR control (**Figure 1C**). In addition, mRNA levels of pro-inflammatory cytokines including TNF-α and IL-6 were elevated (**Figures 1D, E**), indicating the presence of microglia/macrophage polarization and neuroinflammation in the OIR retinas.

### FA inhibited pathological neovascularization in the OIR retina

**Figure 2:**
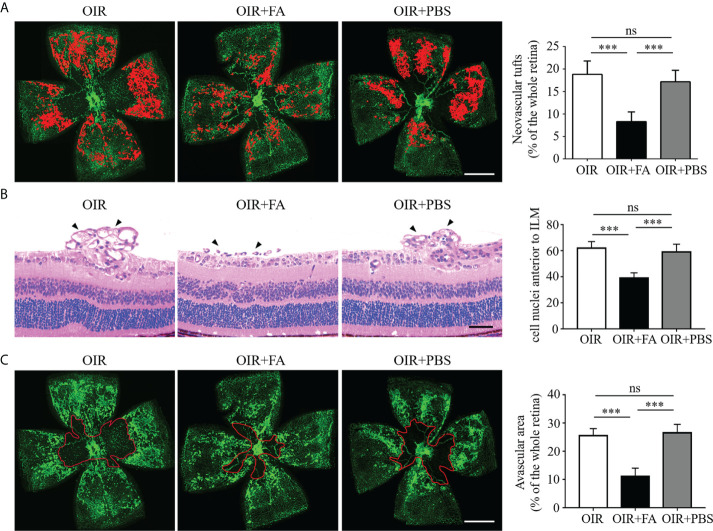
Anti-neovascular effect of FA in the OIR retina. **(A)** Representative images of isolectin-B4 staining on retinal whole-mounts showing neovascular tufts (painting in red). The area of neovascular tufts in the FA-treated group was significantly decreased compared with the untreated and PBS-treated groups. n = 10 mice. Scale bar, 1 mm. **(B)** H&E staining revealed less neovascular cells anterior to the ILM (black arrows) after FA treatment. n = 6 mice. Scale bar, 100 μm. **(C)** Retinal whole-mounts showed less avascular area (circled in red) in the FA-treated group than the untreated and PBS-treated groups. n = 10 mice. Scale bar, 1 mm. Data are presented as mean ± SEM. ***P <0.001, ns, no significance. ILM, inner limiting membrane.

To explore the therapeutic effect of FA on retinal neovascularization, FA was administered intragastrically twice daily from P12 to P17. Retinal neovascularization was assessed using IB4 staining on retinal whole-mounts. Strikingly, the FA-treated retinas presented decreased area of neovascular clusters as compared to PBS-treated ones (**Figure 2A**). The anti-angiogenic effect of FA was also assessed by quantifying the number of endothelial nuclei beyond the internal limiting membrane on retinal sections. Consistently, FA supplement significantly reduced the number of neovascular endothelial cells (**Figure 2B**). In addition, a decrease in avascular area upon FA treatment was observed, indicating a potential effect of FA on promoting reparative angiogenesis (**Figure 2C**). Taken together, these data provide substantial evidence for the therapeutic effect of FA against experimental retinal neovascularization.

### FA skewed M1 microglia/macrophage polarization towards M2 phenotype and orchestrated the inflammatory response

**Figure 3:**
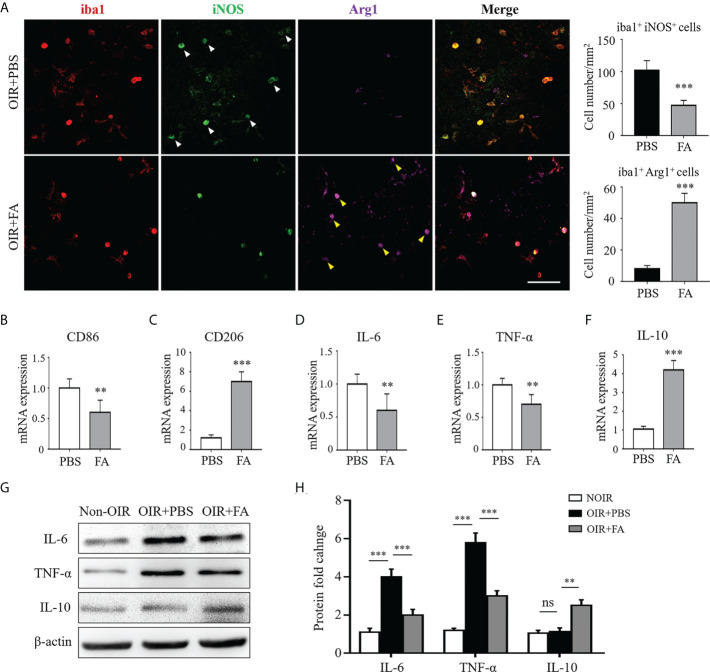
FA switched microglia/macrophage phenotypes from a pro-inflammatory “M1” to an anti-inflammatory “M2”. **(A)** Immunostaining on retinal whole-mounts showed that iba1^+^ microglia/macrophages expressed higher levels of iNOS (white arrowheads) rather than Arg1 in the OIR retina. Note that FA treatment led to an increased number of Arg1^+^ iba1^+^ cells (yellow arrowheads) but decreased number of iNOS^+^ iba1^+^ cells. Scale bar, 100 μm. N = 6 mice. **(B, C)** Real-time PCR showed that FA supplement down-regulated CD86 expression **(B)** while increased CD206 mRNA levels **(C)**. **(D–F)** Pro-inflammatory IL-6 **(D)** and TNF-α **(E)** were decreased while anti-inflammatory IL-10 **(F)** was significantly up-regulated after FA treatment. n = 6 mice. **(G, H)** Western blot assay showed that FA treatment decreased levels of IL-6 and TNF-α, whereas increased the expression of IL-10 in the OIR retinas. n = 6 mice. Data are presented as Mean ± SEM. **P <0.01, ***P <0.001, ns, no significance.

We next evaluated whether FA could regulate microglia/macrophage polarization during pathological neovascularization. In the OIR retinas, the activated microglia/macrophage were almost M1 instead of M2 polarized. FA administration reduced the number of iNOS^+^ microglia/macrophage while increased Arg1 expression, implicating its ability to switch microglia/macrophage polarization from M1 to M2 (**Figure 3A**). Consistently, the RNA level of another M1 marker, CD86, was significantly down-regulated, whereas CD206, another M2 marker, were upregulated with FA treatment (**Figures 3B, C**). In addition, FA suppressed the expression of pro-inflammatory IL-6 and TNF-α while enhanced the expression of anti-inflammatory IL-10 on both mRNA and protein levels (**Figures 3D–H**). These results suggested that FA could trigger microglia/macrophage polarization towards M2 phenotype and attenuated the inflammation cascade in the OIR retina.

### FA drove M1-to-M2 polarization in hypoxia-stimulated BV2 microglia

**Figure 4:**
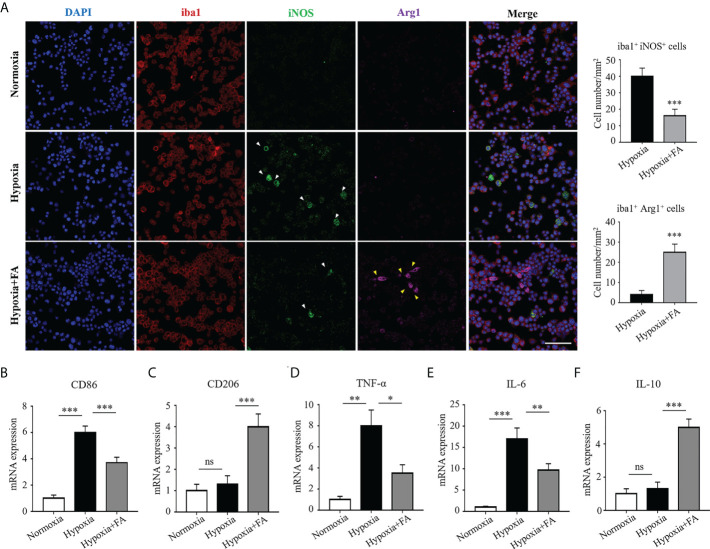
FA orchestrated microglia/macrophage polarization towards an M2 phenotype in cultured BV2 microglial cells. **(A)** Immunofluorescence staining showed increased number of iba1^+^ iNOS^+^ cells in the hypoxia group than normoxia control, whereas FA administration led to reduced iba1^+^ iNOS^+^ cells but increased iba1^+^ Arg1^+^ cells. Scale bar, 100μm. **(B, C)** qRT-PCR assay showed that the mRNA level of CD86 (M1 marker) was up-regulated while CD206 (M2 marker) was down-regulated under hypoxic condition. FA treatment reversed these alternations by increasing CD206 but decreasing CD86 expression. **(D–F)** Levels of pro-inflammatory TNF-α **(D)** and IL-6 **(E)** were increased and anti-inflammatory IL-10 **(F)** was decreased in response to hypoxic stimulus, which were abolished by FA treatment. n = 6 mice. Data are presented as mean ± SEM. *P <0.05, **P <0.01, ***P <0.001, ns, no significance.

To further clarify the regulatory effect of FA on microglia/macrophage polarization, we conducted *in vitro* experiment using cultured BV2 microglial cells under hypoxia condition. The results showed that hypoxia-stimulated BV2 cells exhibited a distinct M1 phenotype, characterized by increased the level of iNOS, as compared with the normoxia group. It was noted that FA treatment significantly impaired iNOS expression while increased the number of Arg1^+^ M2-polarized cells (**Figure 4A**). In addition, CD86 and CD206, markers for M1 and M2 respectively, were also changed from M1-to-M2 activities upon FA treatment (**Figures 4B, C**). Furthermore, the hypoxia insults triggered proinflammatory TNF-α and IL-6 expression in BV2 cells, which could be largely abolished by FA administration (**Figures 4D, E**). FA also significantly increased the level of anti-inflammatory IL-10 in hypoxia-treated cells (**Figure 4F**). These data confirmed the *in vitro* effect of FA on orchestrating microglia polarization and inflammation.

### FA-treated microglial supernatants suppressed tube formation and migration of retinal endothelial cells *in vitro*

**Figure 5:**
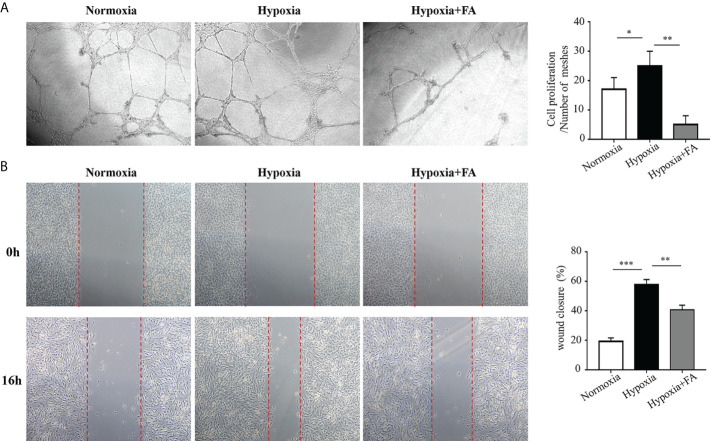
Conditional medium from FA-treated microglia inhibited tube formation and migration of human retinal endothelial cells (HRECs). BV2 microglial cells were cultured under hypoxic conditions (1.5% O_2_) with or without FA treatment for 24 h The supernatants from BV2 cells were collected and applied to the culture medium of HRECs for 16 h **(A)** Tube formation assay showed that hypoxia-stimulated supernatants from microglia promoted tube formation, whereas FA-treated microglia-derived medium inhibited HRECs tube formation. **(B)** Wound healing assay showed increased percentage of wound closure rate in the hypoxia-cultured medium group, which was inhibited when FA-treated BV2-derived culture medium was added. Data are presented as mean ± SEM. *P <0.05, **P <0.01, ***P <0.001.

To elucidate whether FA-induced microglia/macrophage polarization contribute to angiogenesis *in vitro*, we collected M1 microglia culture supernatants (MCS) from hypoxia-cultured BV2 cells and M2 MCS from FA-treated BV2 cells and applied them to HRECs cultures. The results showed that M1 MCS promoted tube formation of HRECs while M2 MCS largely reversed this effect (**Figure 5A**). The effect of MCS on HRECs migration was also evaluated using wound closure test, through which M2 MCS was found to partially abolish the effect of M1 MCS on promoting HRECs migration (**Figure 5B**). These results demonstrated that FA-induced microglia phenotypic switch from M1 to M2 could suppressed HRECs tube formation and migration.

### FA inhibited retinal neovascularization in the OIR by blocking the ROS/NF-κB pathway

**Figure 6:**
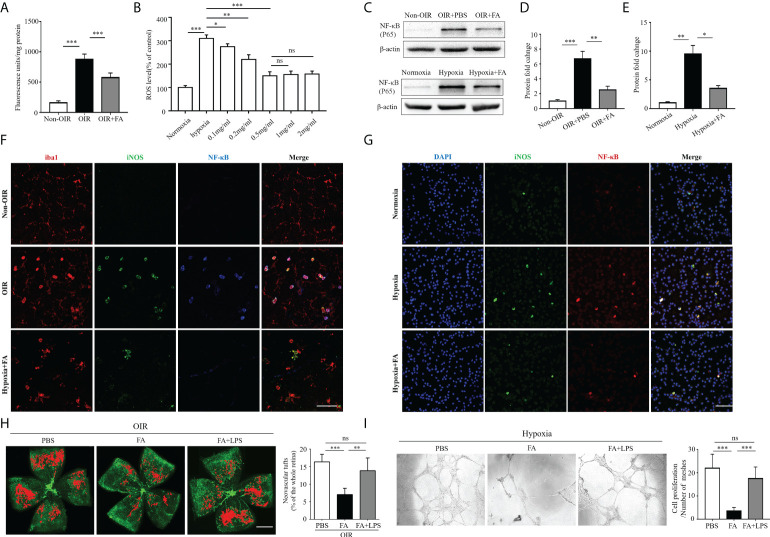
FA exerted anti-angiogenic activities *via* inhibiting the ROS/NF-κB signaling. **(A)** Measurement of ROS levels in the retina under *in vivo* conditions. Retina from OIR and control mice was incubated at 37°C in vials with 5 µM DCFH-DA as described in the method section. The extent of the oxidized fluorescence was measured in each excised retina and presented as fluorescence units/mg of retinal protein. n = 6 mice in each group. **(B)** DCFH-DA was used to detect ROS levels in cultured BV2 cell lines in hypoxic conditions. The results were expressed as percentage of change in fluorescence and the control group was taken as 100%. For treatment, FA was added to the culture medium with different concentrations. **(C–E)** Western blot showed that protein levels of NF-κB (p65) was up-regulated in both OIR retina **(C, D)** and hypoxia-stimulated BV2 cells **(C, E)**, which could be significantly reversed with FA administration. **(F, G)** Immunostaining on retinal wholemounts **(F)** and cultured BV2 cells **(G)** showing co-labeling of iNOS and NF-κB in iba1^+^ cells under hypoxic condition. Note that FA treatment significantly decreased the number of iNOS^+^ NF-κB^+^ cells. Scale bar, 100 μm. n = 6 mice. **(H)** Isolectin-B4 staining on retinal whole-mounts showed that the combination of LPS treatment abolished the anti-neovascular effect of FA in the OIR retina. Scale bar, 1 mm. n= 10 mice. **(I)** Human retinal endothelial cells (HRECs) tube formation assay revealed that co-treatment of LPS with FA significantly reversed the effect of single FA treatment on inhibition of HRECs tube formation. Data are presented as mean ± SEM. *P <0.05, **P <0.01, ***P <0.001, ns, no significance.

Recent data suggest that reactive oxygen species (ROS) play an initial role in driving M1 macrophage polarization (16, 17). Nuclear factor kappa-light-chain-enhancer of activated B cells (NF-κB) is one of the key regulatory molecules in oxidative stress-induced cell activation (18). We hypothesized that FA may attenuate retinal neovascularization by modulating microglia/macrophage phenotypic switching through activating the ROS/NF-κB pathway. To address this hypothesis, we first detected the antioxidant property of FA in both OIR retina and hypoxia-treated BV2 cells. Expectedly, ROS levels were approximately six times higher in the OIR retina than the non-OIR control (**Figure 6A**), and were about three times higher in the hypoxia-treated cells than the normoxia control (**Figure 6B**). Notably, ROS levels were significantly down-regulated with FA treatment, among which the concentration of 0.5mg/ml had the optimal *in vitro* treatment effect (**Figures 6A, B**). The protein level of NF-κB was also significant decreased by FA treatment in both OIR retina and hypoxia-stimulated BV2 cells (**Figures 6C–E**). In addition, NF-κB was perfectly co-labeled with iNOS in both OIR retina (**Figure 6F**) and cultured BV2 microglial cells (**Figure 6G**), indicating a link between NF-κB activation and M1 polarization. FA was able to downregulate NF-κB levels under hypoxic conditions both *in vivo* and *in vitro* (**Figures 6F, G**). We further investigated whether FA worked through inhibiting the ROS/NF-κB signaling by combining FA treatment with intraperitoneal injection of LPS, a well-known activator of NF-κB. As a result, LPS administration largely abolished the anti-angiogenic effect of FA (**Figure 6H**). Consistently, incubation of HRECs with M2 MCS derived from FA-treated BV2 cells induced a significant decrease in HRECs tube formation, whereas co-treatment of FA and LPS largely reversed this effect (**Figure 6I**). The regulatory effect of FA on angiogenic factors including VEGF, platelet derived growth factor (PDGF), fibroblast growth factor-2 (FGF2), and hypoxia-inducible factor 1-α (HIF1α) was also investigated. FA supplement was found to down-regulate VEGF level while had no significant impact on other molecules (**Supplementary Figure 2**). These findings showed that FA inhibited retinal neovascularization mainly through inhibiting the ROS/NF-κB pathway.

### FA supplement had no obvious retinal or systemic side effects in mice

**Figure 7:**
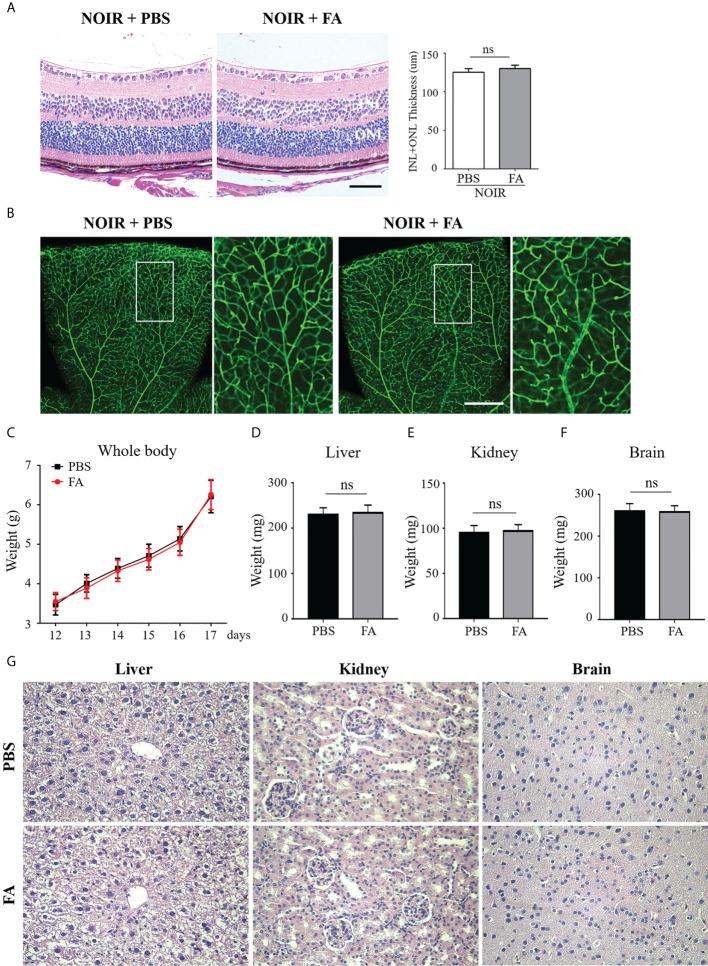
FA treatment had no visible retinal or systemic side-effects. FA (50 mg/kg) or vehicle (phosphate buffered saline, PBS) was administered intragastrically twice daily from P12 to P17. **(A)** H&E staining showing retinal structure and thickness in the FA- and PBS-treated mice. Both groups had well-organized retinal structure. No significant difference in total thickness of INL (inner nuclear layer) and ONL (outer nuclear layer) between the two groups was observed. n=6 mice. Scale bars: 50 μm. **(B)** Assessment of retinal vessels using Isolectin B4 staining on retinal flatmounts. No significant changes in vascular morphology with FA treatment were observed. Scale bar: 500 μm. **(C)** The body weight of OIR mice was measured daily from P12 to P17. No significant difference between FA- and PBS-treated groups was found. n=10 mice. **(D–F)** On P17, the weight of vital organs including liver, kidney, and brain, had no significant differences between the two groups. n=10 mice. **(G)** H&E staining of liver, kidney, and brain tissues in the treatment and control groups. No obvious damage of these organs was noticed in the FA-treated mice. n=6 mice. ns, no significance.

We next investigated the potential toxicity of FA. H&E staining was first performed in which both FA- and PBS-treated non-OIR retina presented well-organized structure and nearly equal thickness of the inner and outer nuclear layers(**Figure 7A**). In addition, FA treatment did not affect the morphology and density of retinal vasculature as compared with PBS-treated control (**Figure 7B**), indicating no potential retinal toxicity of FA. To determine the systemic safety of FA, the mead weight of whole-body as well as vital organs including the liver, kidney, and brain, were measured. The results showed that FA treatment did not lead to significant changes in the weight of body (**Figure 7C**) and vital organs (**Figures 7D–F**). In addition, there was no significant toxicity in the major organs of mice treated with FA compared to the PBS group (**Figure 7G**). These results suggest that FA treatment did not induce significant retinal and systemic side effects in the OIR mice.

## Discussion

Development of VEGF-independent anti-angiogenesis approaches is desired for ocular neovascular diseases, while targeting microglia/macrophage-based neuroinflammation presents a unique immunotherapeutic strategy. In this study, we revealed that activated microglia/macrophage switched towards an “M1” polarized state and fueled the inflammatory response to promote hypoxia-induced pathological retinal angiogenesis. In addition, skewing microglia/macrophage polarization into an anti-inflammatory “M2” phenotype by FA supplement markedly attenuated retinal neovascularization and neuroinflammation, suggesting a promising pharmacological approach for retinal neovascularization.

The role of microglia polarization/macrophage in inflammatory and neurodegenerative diseases has gained increasing attention in recent years. Activated microglia/macrophage are considered to comprise a heterogeneous population of cells and form a continuum state containing two extreme phenotypes called “M1” and “M2”, which is possibly the reason for the duality of microglia/macrophage functions (19). Microglia/macrophage-mediated neuroinflammation played an important part in central nervous system disorders including ischemic stroke, Parkinson’s disease, and Alzheimer’s disease (20, 21). Generally, the pro-inflammatory factors of the M1 phenotype aggravate cell death and neurologic symptoms, whereas the M2 phenotype exerts beneficial effects through the ability to engulf debris and promote the repair and regeneration of the brain tissue (22, 23). Modulation of microglia/macrophage polarization towards a protective phenotype exerted therapeutic effects on these inflammatory and neurodegenerative diseases (24).

In the retina, based on the concept of inflammation-induced angiogenesis, increasing evidence has now indicated the role of microglia/macrophage activation and polarization during retinal neovascularization (25). In an animal model of ischemic retinopathy, a distinct switching towards the M1 microglia/macrophage phenotype was once reported (26). In addition, the time phase of M1/M2 shifting is corresponded with the time phase of regression of neovascular tufts (27), indicating a close relation between microglia/macrophage polarization and neovascularization. Consistent with previous studies, our study found increased expression of iNOS, a well-known M1 marker, in the OIR retina, which mainly located in the ameboid microglia/macrophage. additionally, we detected a small number of M2 microglia/macrophage in both OIR and non-OIR retina, indicating that M2 was not a resting phenotype but an alternatively activated one with anti-inflammation and immunoregulation properties.

Targeting microglia/macrophage polarization using potent immunoregulators develops an alternative way to anti-neovascularization. First found from Ferula foetida, FA is a natural phenolic phytochemical generally present plants and serves as an active ingredient of several Chinese medicinal herbs including Ligusticum wallichii, Angelica sinensis, and Cimicifuga racemose (28). FA is well known for its powerful antioxidant activities which may help fight the effects of free radicals, the skin’s tone, and reduce signs of aging (29). In addition, recent studies have expanded the biological functions of FA, including anti-diabetes, anti-carcinogenesis, anti-apoptosis, and anti-inflammation, which further help in treating various pathophysiological conditions such as hypoxia, cancer, diabetes, neurodegenerative disorders, and viral infections (30–32). The therapeutic value of FA in retinal neovascular diseases remains unclear. In this study, FA exhibited potent anti-angiogenic effect in both *in vivo* and *in vitro* experimental models. In addition, treatment of FA reduced the avascular area in the OIR retina, indicating its potential of promoting vascular normalization and reparative angiogenesis. Mechanically, the anti-angiogenic effect of FA was based on its ability to regulate microglia/macrophage polarization towards an anti-inflammatory M2 phenotype.

Polarized microglia/macrophage exert different functions by secreting a variety of cytokines and chemokines. TNF-α and IL-6 are generally released by classic M1 microglia, which provoke the extravasation of circulating monocytes and granulocytes resulting in vascular damage (33). On the other hand, M2-type cytokines, including IL-10 and TGF-β, are found to prevent tumor-associated angiogenesis by inhibiting reactive oxygen species in endothelial cells (34, 35) and antagonizing the secretion and activities of pro-inflammatory and pro-angiogenic molecules, including TNF-α, IL-6, IL-1β, matrix metalloproteinase (MMP)-9, and VEGF (36, 37). In this study, FA significantly elevated the level of IL-10 in the OIR retina, suggesting that IL-10-expressing M2 microglia/macrophage might be a therapeutic target for retinal anti-angiogenesis.

The precise mechanism of microglia/macrophage polarization during retinal neovascularization remains unclear, although some transcriptional factors showed the potential of modulating microglial polarization during ischemic stroke (38). Based on the antioxidation and anti-inflammation properties of FA, we detected several inflammation- and oxidation-related transcriptional factors and found that NF-κB was strikingly downregulated upon FA treatment, both *in vivo* and *in vitro*. NF-κB is a widespread and heterodimeric nuclear transcription factor participating in multiple physiological and pathological processes through binding to cytokines, gene promoters or enhancers, and immunomodulators (39). Activation of the ROS/NF-κB axis was once demonstrated to play a part in oxidative stress-induced cell activation during tumorigenesis (40). More importantly, FA has been recognized as a protector against iron toxicity and ROS production. Under ischemic conditions, a large amount of iron is released, which catalyzes the production of more free radicals. Iron oxidation through the Fenton reaction and the iron-catalyzed Haber-Weiss reaction generate highly ROS, such as the hydroxyl radical, while FA was able to protect the retina against this iron toxicity by acting as a free radical scavenger (41–43). In this study, we found that both ROS and NF-κB was upregulated in the OIR retina, which could be largely abolished by FA treatment, indicating that FA attenuated retinal angiogenesis at least in part through inhibiting the ROS/NF-κB signaling. In addition, consistent with a previous study reporting a possible link between NF-κB and macrophage polarization (44), our data using immunostaining showed that NF-κB and iNOS were strictly co-localized in the OIR, implying a regulatory effect of NF-κB among hypoxia insults, microglia/macrophage polarization, and inflammation. Further study using NF-κB conditional knockout mice is essential to clarify the exact role of ROS/NF-κB signaling during retinal neovascularization and its therapeutic potential.

A recent study showed that FA could have toxic effects on *in vitro* intestinal cell models at high concentrations, suggesting that an excessive and uncontrolled consumption should be avoided (45). In this study, FA was administered intragastrically twice daily at a concentration of 50 mg/kg, which was considered a safe dose (45). In addition, we did not find any local or systemic side effects in both animals and cultured retinal endothelial cells, which further confirmed the safety of FA administration in this study.

In conclusion, using *in vitro* and *in vivo* experimental models, our data showed that FA, a natural and active extract from plant, orchestrated microglia/macrophage polarization from a pro-inflammatory M1 phenotype towards an anti-inflammatory M2 phenotype and played potent anti-angiogenic effect in hypoxia-induced angiogenesis. In addition, the ROS/NF-κB axis was involved in the FA-mediated microglia/macrophage polarization and anti-angiogenesis, supporting ROS/NF-κB as a potential therapeutic target for the treatment of retinal neovascular diseases.

## Data availability statement

The original contributions presented in the study are included in the article/**Supplementary Material**. Further inquiries can be directed to the corresponding author.

## Ethics statement

All animal studies adhered to guidelines for the care and use of laboratory animals issued by the National Institutes of Health (NIH) and were reviewed and approved by the local Animal Ethic Committee of Animal Laboratories of Yantai Yuhuangding Hospital. Mice were anesthetized with intramuscular injection of zoletil and xylazine. Body temperature was maintained at 37 ± 0.5°C during surgery. At the end of the experiments, mice were euthanized through carbon dioxide asphyxiation of air with 100% carbon dioxide.

## Author contributions

ZH and YL contributed to the conception and design of the experiments. XS, LM, XL, and JW were responsible for data collection, analysis, and interpretation. ZH and XS drafted the article. XS, LM and YL contributed to the data analysis. ZH revised the article. All authors approved the final version of the manuscript for submission. All authors contributed to the article and approved the submitted version.

## Funding

This work was supported by the National Natural Science Foundation of China (No. 82101112) to ZH.

## Conflict of interest

The authors declare that the research was conducted in the absence of any commercial or financial relationships that could be construed as a potential conflict of interest.

## Publisher’s note

All claims expressed in this article are solely those of the authors and do not necessarily represent those of their affiliated organizations, or those of the publisher, the editors and the reviewers. Any product that may be evaluated in this article, or claim that may be made by its manufacturer, is not guaranteed or endorsed by the publisher.

## Supplementary material

Supplementary Figure 1 Establishment of the Oxygen-induced retinopathy (OIR) murine model. Mouse pups were exposed to 75% oxygen from postnatal day 7 (P7) to P12 using an Oxy Cycler system and then returned to room air. Retinas at different timepoints (P12, P13, P14, P15, P16, P17, P21, P25) were collected, stained with Isolectin B4, and flat-mounted. The images were obtained using a microscope (Leica DM4000, Germany). Non-OIR retinas (P12, P17) was used as a control.

Supplementary Figure 2 qRT-PCR analysis of proangiogenic factors and HIF-1α in the OIR retinas. Note that the mRNA levels of VEGF, FGF2, PDGF-A, and HIF-1α were up-regulated in the OIR retinas. FA treatment down-regulated VEGF level but had no impact on other molecules. Data are presented as mean ± SEM. **P <*0.05, ***P <*0.01, ns, no significance.

## Abbreviations

CCL, CC chemokine ligand; HRECs, human retinal endothelial cells; IB4, isolectin-B4; IL, interleukin; iNOS, inducible nitric oxide synthase; LPS, lipopolysaccharide; MCS, microglia culture supernatants; NF-κB, Nuclear factor kappa-light-chain-enhancer of activated B cells; OIR, oxygen-induced retinopathy; PCR, polymerase chain reaction; ROS, reactive oxygen species; TGF, transforming growth factor; TNF, tumor necrosis factor; VEGF, anti-vascular endothelial growth factor.
