# Supplementary figures and images for: Ferulic acid alleviates retinal neovascularization by modulating microglia/macrophage polarization through the ROS/NF-κB axis
Source: Front Immunol. 2022 Sep 2;13:976729. doi: 10.3389/fimmu.2022.976729 (PMC9478033; doi:10.3389/fimmu.2022.976729)

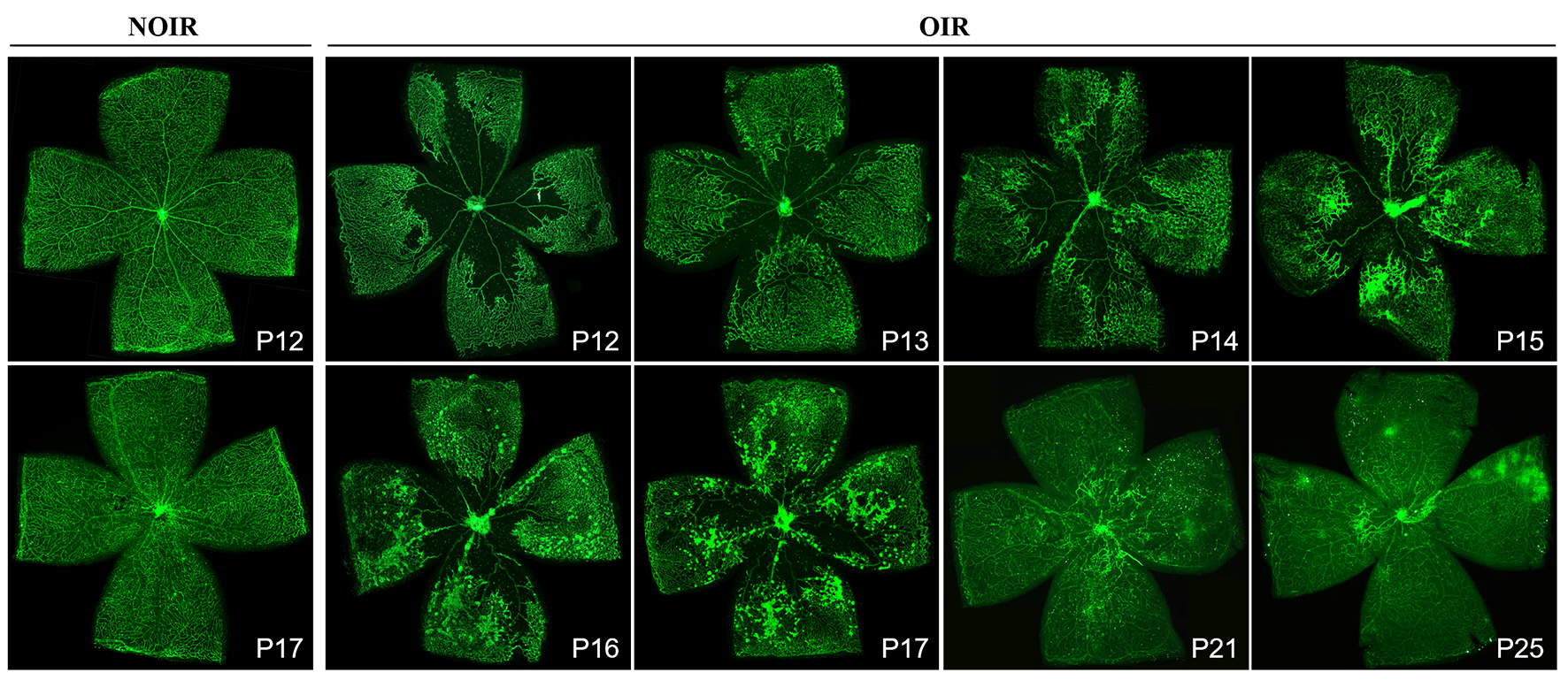

Supplement: Supplementary Figure 1 — Establishment of the Oxygen-induced retinopathy (OIR) murine model. Mouse pups were exposed to 75% oxygen from postnatal day 7 (P7) to P12 using an Oxy Cycler system and then returned to room air. Retinas at different timepoints (P12, P13, P14, P15, P16, P17, P21, P25) were collected, stained with Isolectin B4, and flat-mounted. The images were obtained using a microscope (Leica DM4000, Germany). Non-OIR retinas (P12, P17) was used as a control. [file Image_1.tif]

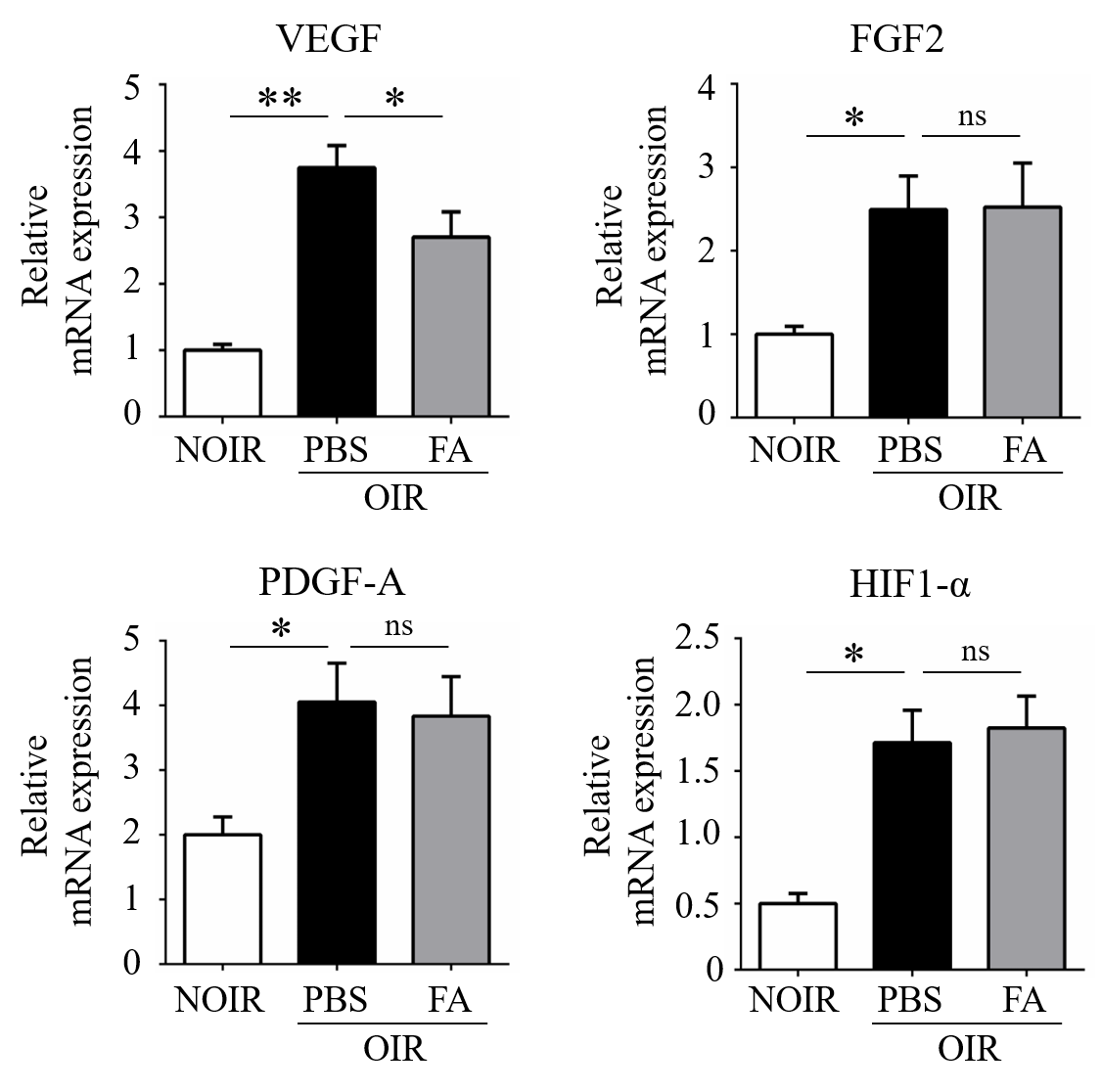

Supplement: Supplementary Figure 2 — qRT-PCR analysis of proangiogenic factors and HIF-1α in the OIR retinas. Note that the mRNA levels of VEGF, FGF2, PDGF-A, and HIF-1α were up-regulated in the OIR retinas. FA treatment down-regulated VEGF level but had no impact on other molecules. Data are presented as mean ± SEM. *P <0.05, **P <0.01, ns, no significance. [file Image_2.tif]
